# Transforming Growth Factor-β and Interleukin-10 Synergistically Regulate Humoral Immunity *via* Modulating Metabolic Signals

**DOI:** 10.3389/fimmu.2018.01364

**Published:** 2018-06-14

**Authors:** Toshihiko Komai, Mariko Inoue, Tomohisa Okamura, Kaoru Morita, Yukiko Iwasaki, Shuji Sumitomo, Hirofumi Shoda, Kazuhiko Yamamoto, Keishi Fujio

**Affiliations:** ^1^Department of Allergy and Rheumatology, Graduate School of Medicine, The University of Tokyo, Tokyo, Japan; ^2^Department of Functional Genomics and Immunological Diseases, Graduate School of Medicine, The University of Tokyo, Tokyo, Japan; ^3^Max Planck-The University of Tokyo Center for Integrative Inflammology, The University of Tokyo, Tokyo, Japan; ^4^Laboratory for Autoimmune Diseases, Center for Integrative Medical Sciences, RIKEN, Kanagawa, Japan

**Keywords:** humoral immunity, systemic lupus erythematosus, cytokine synergy, transforming growth factor-β, interleukin-10, B cells, immunometabolism

## Abstract

Inhibitory cytokines, such as transforming growth factor-β (TGF-β) and interleukin-10 (IL-10), are humoral factors involved in the suppressive function of regulatory T cells and play critical roles in maintaining immune homeostasis. However, TGF-β and IL-10 also have pleiotropic effects and induce humoral immune responses depending on conditions, and thus their therapeutic application to autoimmune diseases remains limited. Here, we show that a combination of TGF-β and IL-10, but not single cytokine, is required to suppress B cell activation induced by toll-like receptor (TLR) stimulation. In *in vivo* analyses, the simultaneous presence of TGF-β and IL-10 effectively suppressed TLR-mediated antigen-specific immune responses and ameliorated pathologies in imiquimod (TLR7 agonist)-induced lupus model and lupus-prone MRL/*lpr* mice. Intriguingly, TGF-β and IL-10 synergistically modulated transcriptional programs and suppressed cellular energetics of both glycolysis and oxidative phosphorylation *via* inhibition of the mammalian target of rapamycin complex 1 (mTORC1)/S6 kinase 1 (S6K1) pathway in TLR-stimulated B cells. On the other hand, enhancement of mTOR signaling and mitochondrial biosynthesis in TLR-stimulated B cells counteracted the synergistic inhibitory effects. The inhibitory cytokine synergy of TGF-β and IL-10 *via* suppression of energy metabolism was also observed in human TLR-stimulated B cells. There is increasing evidence supporting the importance of adequate metabolic signals in various immune cells to exert their immune function. In this study, we have shown that a previously unrecognized synergy of inhibitory cytokines regulates systemic humoral immune responses *via* modulating immunometabolism in B cells. Our findings indicate that inhibition of B cell metabolism mediated by two synergistic cytokines contributes to the induction of immune tolerance and could be a new therapeutic strategy for autoimmune diseases such as systemic lupus erythematosus.

## Introduction

The balance of cytokines controls immune responses and is implicated in the pathogenesis of autoimmune diseases (1). Inhibitory cytokines like transforming growth factor-β (TGF-β) (2–4) and interleukin-10 (IL-10) (5, 6) are key regulators of immune homeostasis, as mice deficient for either of these regulatory cytokines develop severe inflammatory diseases (7, 8). Blockade of pro-inflammatory cytokines offers effective treatment of inflammatory autoimmune diseases such as rheumatoid arthritis (9, 10). However, therapeutic effects of blockade of pro-inflammatory cytokines for other systemic autoimmune diseases, such as systemic lupus erythematosus (SLE), are limited (11). Regulatory T cells (Tregs) are a subpopulation of T cells, which play an essential role in maintaining immunological self-tolerance, and their immunoregulatory mechanisms have been vigorously investigated and considered for treatment application in recent years (2). Inhibitory cytokines are one of the major contributors to immune tolerance by Tregs but therapeutic application of inhibitory cytokines remains limited; difficulties in use are due to their pleiotropic and context-dependent effects (12, 13).

The pathogenesis of SLE is supposed to be a sustained loss of self-tolerance and autoantibody production contributed by genetic and environmental factors (14). Among immune cells, B cells are centrally involved in the immunopathogenesis of SLE (15). Only belimumab, an anti-B cell activating factor (BAFF), is a targeted biologic agent for SLE approved by the US Food and Drug Administration (14, 15). B cells are one of the primary targets for regulating autoimmune diseases because high-affinity antibodies are produced through interaction of germinal center B (GCB) cells with follicular helper T (T_FH_) cells (16, 17). Also, extrafollicular B cell activation by sensing nucleic acid *via* innate receptors, including toll-like receptors (TLRs), contributes to the production of autoantibodies and the pathogenesis of SLE (18, 19).

The effects of representative inhibitory cytokines, TGF-β, and IL-10, on B cells have been examined. Physiological importance of signaling from TGF-β family cytokines in T cells has been elucidated using *Tgfbr2^fl/fl^*CD4-Cre^+^ mice which show T_FH_ cell accumulation and self-reactive B cell activation (20). We recently revealed that TGF-β3 produced by CD4^+^CD25^−^LAG3^+^ Tregs (LAG3^+^ Tregs) is required for the adequate control of humoral immunity (3, 21, 22), indicating immunological roles of TGF-β3 in immune tolerance. On the other hand, the direct effects of IL-10 on B cells are reported to be stimulatory (12), although IL-10 exerts a suppressive function on T_FH_ cells (23). Cytokines display diverse functions in combination with each other, and the combined response of multiple cytokines, which has greater effects than the sum of its parts, is termed cytokine synergy (24). However, synergistic effects of TGF-β and IL-10 in humoral immunity have not yet been investigated.

In this study, we have investigated the roles of TGF-β and IL-10 on humoral immune responses. We examined the effects of these cytokines on B cells from mice or humans under TLR stimulation *in vitro*. Further, therapeutic potentials of these inhibitory cytokines on a mouse model of SLE are also verified. Our findings of synergistic regulation of humoral immunity by TGF-β and IL-10 *via* regulating cellular metabolism provide novel insights for clinical applications of inhibitory cytokines on autoimmune diseases.

## Materials and Methods

### Mice

BALB/c, C57BL/6J (B6), MRL-Fas^+/+^ (MRL/+), and MRL-Fas*^lpr/lpr^* (MRL/*lpr*) mice were purchased from Japan SLC (Hamamatsu, Japan). Green fluorescent protein (GFP)-microtubule-associated protein 1A/1B-light chain 3 (GFP-LC3) mice (25) were kindly provided by N. Mizushima (The University of Tokyo, Japan).

### Murine B Cell Isolation

Spleens were cut into small pieces and digested with collagenase type IV (Sigma, St. Louis, MO, USA). After hemolysis with ammonium chloride with potassium (ACK) lysis buffer, magnetic separation of B cells was performed using a Mouse B Cell Isolation Kit (Miltenyi Biotec, Bergisch Gladbach, Germany), according to the manufacturer’s protocol. Cells were cultured in RPMI 1640 medium supplemented with 10% fetal bovine serum (FBS, BioWest, Nuaillé, France), 100 μg/ml l-glutamine, 100 U/ml penicillin, 100 µg/ml streptomycin (Invitrogen, Carlsbad, CA, USA), and 50 µM 2-mercaptoethanol (2-ME, Sigma).

### Antibody Production

Splenic 1 × 10^5^ B cells/well in 96-well flat bottom plate were stimulated by 3 µg/ml lipopolysaccharides (LPS from *Escherichia coli* O55:B5, Sigma) with or without 10 ng/ml recombinant (r) TGF-β1 (Miltenyi Biotec), rTGF-β3 (Miltenyi Biotec), and/or 50 ng/ml rIL-10 (R&D Systems, Minneapolis, MN, USA) for indicated days. In some experiments, 1 × 10^5^ B cells/well in 96-well flat bottom plate were stimulated by 10 µg/ml anti-CD40 (BD Bioscience, San Jose, CA, USA) and 20 ng/ml rIL-4 (Cell Signaling, Danvers, MA, USA) or 10 µg/ml LPS with 2 ng/ml TGF-β3 and/or 50 ng/ml rIL-10 as indicated. The following reagents were further or alternatively added in some experiments: 25 ng/ml IL-6 (R&D Systems), 1 ng/ml IL-17 (R&D Systems), 200 ng/ml R-848 (Enzo Life Sciences, Farmingdale, NY, USA), 0.1 mM or 1 mM sodium pyruvate (Invitrogen), 1 mM 2-deoxy-d-glucose (2-DG, Sigma), 0.5 µM antimycin A (AA) (Enzo Life Science), 0.5 µM rotenone (Rot) (Sigma), 10 µM M1 (Merck, Darmstadt, Germany), 10 µM Mdivi1 (Sigma), 2 µM 4,6-dimorpholino-*N*-(4-nitrophenyl)-1,3,5-triazin-2-amine (MHY1485, Sigma), and dimethyl sulfoxide (DMSO, Wako, Osaka, Japan). Total IgG, IgA, IgG1, IgG2b, and IgG3 production in the culture supernatants on day 7 was quantified by mouse IgG, IgA, IgG1, IgG2b, and IgG3 ELISA Quantitation Set (Bethyl Laboratories, Montgomery, TX, USA).

### Flow Cytometric (FCM) Analysis

After blocking Fc receptors with anti-CD16/CD32 Ab (BD Bioscience), surface staining of isolated single cells was performed in PBS with 2% FBS with the following monoclonal antibodies (mAb) or reagent: PE anti-CD138 (282-2, BioLegend; 1:100 dilution), APC-Cy7 anti-B220 (RA3-6B2, BioLegend; 2:100), 7-AAD (BioLegend; 3:100), FITC anti-GL-7 (GL7, BD Bioscience; 0.5:100), PE anti-PD-1 (J43, BD Bioscience; 1.5:100), APC anti-Fas (15A7, eBioscience), biotinylated mAb for CD8a (53-6.7, BioLegend), CD11c (N418, BioLegend), CD19 (1D3, eBioscience), and CXCR5 (2G8, BD Bioscience; 5:100), and streptavidin (SA)-APC (BioLegend; 1.5:100). For phosphorylated-protein detection, cells were stained with p-Ser235.236-S6 ribosomal protein (D57.2.2E, Cell Signaling; 1:100) and p-Thr37/46-4E-BP1 (236B4, Cell Signaling; 1:100) after preparing with BD Phosflow lyse/fix buffer and perm buffer III (BD Bioscience), according to the manufacturer’s protocol. For MitoTracker staining, cultured cells stained with 30 nM MitoTracker Green (Invitrogen) and 30 nM MitoTracker DeepRed (Invitrogen) were incubated in a CO_2_ incubator at 37°C for 30 min, according to the manufacturer’s protocol. Stained cells were analyzed and sorted using a FACSVantage SE (Becton-Dickinson, Franklin Lakes, NJ, USA). Data were further assessed with FlowJo (Tree Star, Ashland, OR, USA).

### Human B Cell Isolation and Culture

Peripheral blood mononuclear cells were separated by density-gradient centrifugation with Ficoll-Paque PLUS (GE Healthcare, Little Chalfont, UK). After treatment with ACK lysis buffer, magnetic separation of B cells was performed using Human B Cell Isolation Kit II (Miltenyi Biotec), according to the manufacturer’s protocol. Cells were cultured in serum-free AIM-V medium (ThermoFisher Scientific, Waltham, MA, USA) containing l-glutamine, 50 µg/ml streptomycin sulfate, 10 µg/ml gentamicin sulfate, and Albumax. Isolated B cells (1 × 10^5^) stimulated by 2.5 µg/ml CpG-ODN2006 (Enzo Life Sciences), 1,000 U/ml IL-2 (R&D Systems), 10 ng/ml IL-6 (BioLegend, San Diego, CA, USA), and 0.5 µg/ml anti-CD40 antibody (eBioscience, San Diego, CA, USA) were cultured with the indicated concentration of rTGF-β3 and/or 10 ng/ml IL-10 (PeproTech, Rocky Hill, NJ, USA). In some experiments, 1 µg/ml anti-IL-10 antibody (R&D Systems) was used.

### Cell Proliferation Assay

Isolated splenic B cells were labeled with 0.2 µg/ml 5-(and 6-) carboxyfluorescein diacetate succinimidyl ester (CFSE; Dojindo, Kumamoto, Japan) at 37°C for 10 min, and then 3 × 10^5^ B cells/well in 96-well flat bottom plate were stimulated by 10 µg/ml anti-CD40 Ab and 10 µg/ml anti-IgM Ab (Jackson ImmunoResearch Laboratories, West Grove, PA, USA) or 10 µg/ml LPS with 2 ng/ml TGF-β3 and/or 50 ng/ml IL-10 for 3 days. Cells were further stained with anti-B220 mAb and 7-AAD, and viable 7-AAD^−^CFSE-labeled B220^+^ B cells were assessed by flow cytometry.

### Construction and Injection of the Plasmid Vector

Full-length fragments of murine *Il10, Tgfb1*, and *Tgfb3* were subcloned into the pCAGGS vector, which has the CAG (cytomegalovirus immediately early enhancer/chicken β-actin hybrid) promoter, kindly provided by Junichi Miyazaki (Osaka University Medical School, Japan). The recombinant plasmids were transformed into *Escherichia coli* JM109 (Toyobo, Osaka, Japan) and isolated in large scale using the EndFree plasmid Maxi kit (Qiagen, Hilden, Germany) following the manufacturer’s instructions. Purified plasmid DNA (100 µg) of pCAGGS-Mock, pCAGGS-Tgfb1, and pCAGGS-Tgfb3 in 100–200 µl Ringer’s solution was administered i.v. Purified plasmid DNA (50 µg) of pCAGGS-Il10 in 1 ml Ringer’s solution was administered i.v. Serum cytokine protein levels after each vector administration were measured using a mouse latent TGF-β1 ELISA kit (BioLegend) and a mouse latent TGF-β3 ELISA kit (MyBioSource, San Diego, CA, USA), according to the manufacturer’s protocol.

### Injection of Plasmid DNA Into MRL/*lpr* Mice

MRL/*lpr* mice were administered 100 µg pCAGGS-Mock or pCAGGS-Tgfb3 plasmid vectors i.v. every 4 weeks and were analyzed at the age of 21 weeks. Spleen weights were also measured then. Proteinuria was semi-quantitatively assessed using dipsticks (Albustix; Bayer, West Haven, CT, USA). Anti-dsDNA antibody levels in the sera of 21-week-old mice were measured using a mouse anti-dsDNA ELISA Kit (Shibayagi, Gunma, Japan), according to the manufacturer’s protocol. Mice were randomly assigned to specific treatment groups, and to prevent research outcomes from being influenced by observer bias, proteinuria progressions were evaluated by an examiner blind to the experimental conditions.

### Immunizations

Sex- and age-matched C57BL/6J mice were intraperitoneally (i.p.) administered 200 µg NP-KLH (Biosearch Technologies, Novato, CA, USA) emulsified with an equal amount of Imject Alum (Thermo Scientific), 100 µg NP-KLH emulsified with complete Freund’s adjuvants (Sigma), or 20–100 µg NP-LPS (Biosearch Technologies). Mouse sera were collected for ELISA, and isolated splenocytes were analyzed by flow cytometry at day 7 following the immunization. pCAGGS-Il10, pCAGGS-Tgfb1, and/or pCAGGS-Tgfb3 vectors were administered i.v. 1 day before the indicated immunization. Anti-IL-10 mAb (300 µg) (JES5-2A5, Bio X Cell, West Lebanon, NH, USA) and 300 µg anti-TGF-β antibody (1D11.16.8, Bio X Cell) were administered i.p. at day −1, 2, and 5.

### Quantification of NP-Specific Antibody Responses

Anti-NP IgG, IgG2b, IgM, or IgA levels were quantified by ELISA on plates coated with NP8-bovine serum albumin (BSA) (Biosearch Technologies) for the capture antigen. Serially diluted pooled sera from NP-KLH or NP-LPS immunized C57BL/6 mice were utilized as a standard. Following the incubation with sample and control sera, HRP-conjugated goat anti-mouse IgG, IgG2b, IgM, or IgA antibody (Bethyl Laboratories) and further TMP substrate (KPL, Gaithersburg, MD, USA) were added to the plates.

### *In Vivo* Imiquimod Application

The skin on each BALB/c mouse ear was topically treated with 1.25 mg of Beselna cream (5% imiquimod cream, Mochida Pharmaceutical, Tokyo, Japan) 3 times weekly, as described previously (26). Purified 50 µg pCAGGS-Il10 and/or 100 µg pCAGGS-Tgfb3 were administered i.v. every 2 weeks. Serum anti-dsDNA Ab levels were measured using a mouse anti-dsDNA ELISA Kit (Shibayagi) at week 4.

### Fluorescence Microscopy and Detection of GFP-LCII

Splenic B cells from GFP-LC3 mice were cultured for 3 days with the indicated cytokines. Cells were washed with 0.05% saponin for 3 min and fixed for 10 min at room temperature with 4% paraformaldehyde. Images were captured using a fluorescence microscopy instrument (IX83, Olympus, Tokyo, Japan). GFP-positive puncta were counted manually in a blinded manner. For FCM assessment of GFP-LCII, cultured cells were treated with 100 µM chloroquine the last 4 h, washed with 0.05% saponin for 3 min, and analyzed.

### Quantitative Real-Time PCR

Total RNAs were extracted with RNeasy Micro Kit (Qiagen) and were reverse-transcribed to cDNA with random primers (Invitrogen) and SuperScript III (Invitrogen), as described elsewhere (3). Quantitative real-time PCR (qRT-PCR) was performed using CFX Connect Real-Time PCR Detection System (Bio-Rad, Hercules, CA, USA) with QuantiTect SYBR Green PCR Kit (Qiagen). The following forward (FW) and reverse (RV) primer pairs were used: *Xbp1* (FW: AGCAGCAAGTGGTGGATTTG, RV: CCAAGCGTGTTCTTAACTCCT), *Prdm1* (FW: GCCAACCAGGAACTTCTTGTGT, RV: AGGATAAACCACCCGAGGGT), *Bcl6* (FW: GCAGTTTAGAGCCCATAAGA, RV: GTACATGAAGTCCAGGAGGA), *Bach2* (FW: CAGTGAGTCGTGTCCTGTGC, RV: TTCCTGGGAAGGTCTGTGAT), *Myc* (FW: GGACAGTGTTCTCTGCC, RV: CGTCGCAGATGAAATAGG), *P4hb* (FW: CAAGATCAAGCCCCACCTGAT, RV: AGTTCGCCCCAACCAGTACTT), *Dnajb9* (FW: TAAAAGCCCTGATGCTGAAGC, RV: TCCGACTATTGGCATCCGA), *Irf4* (FW: GCCCAACAAGCTAGAAAG, RV: TCTCTGAGGGTCTGGAAACT), *Cox5a* (FW: GGGTCACACGAGACAGATGA, RV: GGAACCAGATCATAGCCAACA), *Cox5b* (FW: ACCCTAATCTAGTCCCGTCC, RV: CAGCCAAAACCAGATGACAG), *Ndufs7* (FW: GCGTGCTGTGCCGTGGAGAT, RV: CGTACACCTTTCGGAGCGCGG), *Atp5d* (FW: CTCCTCTGTGCAGTTACTAGCTGAA, RV: ACTGCGCCTTCTCCAGGTT), and *Actin* (FW: AGAGGGAAATCGTGCGTGAC, RV: CAATAGTGATGACCTGGCCGT). Relative expression was calculated based on control β-actin abundance.

### RNA-Sequencing

Splenic B cells (3.3 × 10^6^) in 24-well plates were stimulated by 10 µg/ml LPS in the presence of 2 ng/ml TGF-β3 and/or 50 ng/ml IL-10 for 3 days. Total RNA of flow cytometrically purified B220^+^7-AAD^−^ B cells from the cultured cells was extracted with RNeasy Mini kit (Qiagen) and libraries were prepared using TruSeq Stranded mRNA HT Sample Prep Kit (Illumina, San Diego, CA, USA) following the manufacturer’s instructions. Sequencing was performed using HiSeq 2500 instrument (Illumina). Sequence reads were mapped to the mouse genome GRCm38/mm10 by TopHat2, and read counts were obtained using HTSeq. The detection of differentially expressed genes (DEGs) measured by the informative/non-informative (I/NI) value more than 0.2 as well as the normalization were performed using the R package Dexus. Hierarchical clustering and heatmap visualization of the DEGs were further performed using the R package Heatplot. DEGs were clustered into 16 groups and pathways in each group were investigated using Inguinal Pathway Analysis software (Qiagen). Fisher’s exact test was used for the calculation of *P*-value determining whether the pathways were enriched with genes of interest.

### Western Blot Analysis

Isolated B cells were stimulated by 3 µg/ml LPS and cultured with or without 10 ng/ml rTGF-β3 and/or 50 ng/ml rIL-10 for 24–48 h. Lysates were prepared with lysis buffer (50 mM Tris–HCl, 0.15 M NaCl, 1% Triton X-100, 1 mM EDTA) and protein concentrations in the cell lysates were measured using a BCA protein assay kit (Pierce, Rockford, IL, USA). Proteins were further denatured with Laemmli sample buffer (Sigma) at 95°C and resolved on Mini-Protean TGX precast gels (Bio-Rad). Following blotting onto polyvinylidene fluoride membranes (Millipore, Billerica, MA, USA) and blocking with 5% BSA, blots were incubated overnight with the following primary antibodies: p-Thr389-p70 S6 kinase (Cell Signaling), p-Thr37/46-4E-BP1 (236B4, Cell Signaling), p70 S6 kinase (49D7, Cell Signaling), 4E-BP1 (53H11, Cell Signaling), HIF-1α (D2U3T, Cell Signaling), PGC-1α (4C1.3, Merck), p-Tyr705-signal transducers and activator of transcription 3 (STAT3) (D3H7, Cell Signaling), p-Ser423/425-Smad3 (C25A9, Cell Signaling), and GAPDH (D16H11, Cell Signaling). The blots were further incubated with secondary anti-rabbit IgG-HRP Ab (Invitrogen) or anti-mouse IgG-HRP Ab (Bethyl Laboratories), and developed with ECL substrate (GE Healthcare), according to the manufacturer’s protocol. The immunoblot signals were detected using ImageQuant LAS4010 (GE Healthcare).

### Extracellular Flux Analysis

An XF96 Extracellular Flux analyzer (Seahorse Bioscience, North Billerica, MA, USA) was used for the quantification of oxygen consumption rate (OCR) and ECAR. OCR was measured in XF media (Agilent Technologies, Santa Clara, CA, USA) supplemented with 1 mM sodium pyruvate and 10 mM glucose and 2 mM l-glutamine (Sigma) under basal condition and in response to 1 µM oligomycin, 2 µM carbonyl cyanide-*p*-trifluoromethoxyphenylhydrazone (FCCP), and 0.5 µM Rot/AA. ECAR was measured in XF media supplemented with 1 mM l-glutamine under basal conditions and in response to 10 mM glucose, 1 µM oligomycin, and 50 mM 2-deoxy-glucose. LPS-stimulated B cells with each cytokine were resuspended in XF media and were plated on XF96 cell culture microplates (1 × 10^5^ cells per well) coated with Cell-Tak (BD Biosciences).

### Statistics

Statistical significance and analysis of variance (ANOVA) between indicated groups were analyzed by GraphPad Prism version 6.0 h (GraphPad Software, Inc., La Jolla, CA, USA). A comparison of more than two group means was analyzed by ANOVA with Bonferroni’s or Holm–Sidak’s multiple comparison tests. A comparison of two group means was analyzed by two-tailed *t*-test except for the semi-quantitative evaluation of proteinuria progression by Mann–Whitney *U* test. Statistically significant differences were considered at *P* < 0.05 for all tests. Data in the figures were expressed as mean ± SD or SEM.

## Results

### Inhibitory Effect of TGF-β on T-Cell-Dependent Humoral Immunity

**Figure 1:**
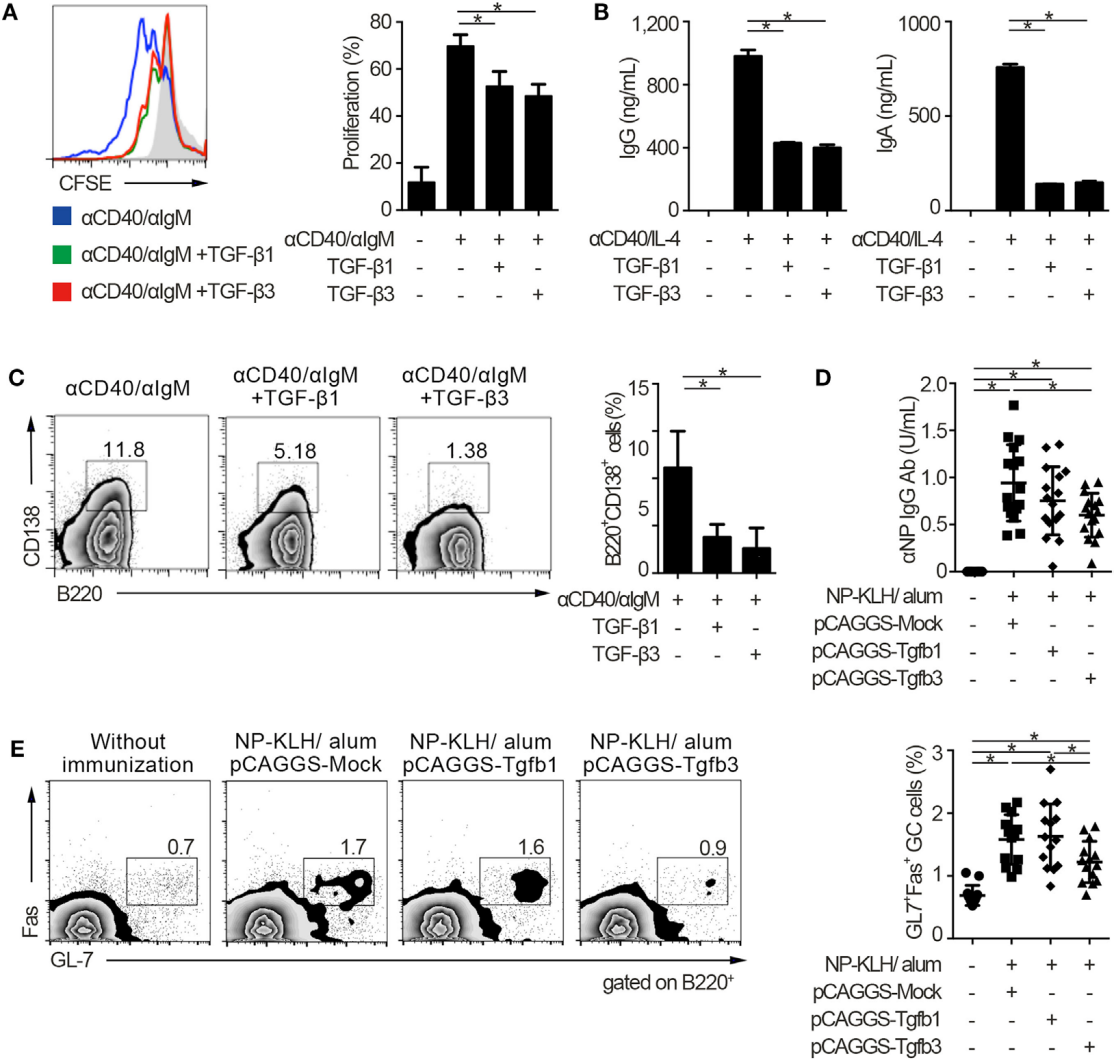
TGF-β regulates T cell-dependent B cell activation. **(A)** Histogram plots of CFSE-labeled B220^+^ B cells stimulated by 10 µg/ml anti-CD40 and 10 µg/ml anti-IgM either with TGF-β1 or TGF-β3 for 3 days (*n* = 3). Data are representative histograms with CFSE fluorescence gated on B220^+^ cells and the percentage of proliferating B220^+^CFSE^−^ cells. **(B)** Total IgG and IgA antibody titers in the supernatants of anti-CD40/IL-4 stimulated B cells with either TGF-β1 or TGF-β3 and 50 ng/ml interleukin-10 for 7 days quantified by ELISA (*n* = 3). **(C)** Flow cytometry analyzing the expression of B220 and CD138 in B cells stimulated with anti-CD40/anti-IgM either with or without TGF-β1 or TGF-β3 for 3 days (left), and quantification of B220^+^CD138^+^ plasmablasts (right) (*n* = 3). Data are representative of three independent experiments in **(A–C)**. **(D)** C57BL/6 (B6) mice treated with indicated pCAGGS plasmid vectors were by i.p. injection of 100 µg NP-KLH in alum. Anti-NP-bovine serum albumin antibody titers in the sera (*n* = 16) were quantified by ELISA 7 days after the immunization. **(E)** Flow cytometric (FCM) plots and quantification of GL-7^+^Fas^+^ germinal center B cells in B220^+^ cells from B6 mice administered the indicated pCAGGS vectors and immunized with 100 µg NP-KLH in alum (*n* = 13–14). *P* < 0.05 by one-way analysis of variance followed by Bonferroni’s multiple comparisons test **(A–C)**, and Holm–Sidak’s multiple comparisons test **(D,E)**. Error bars, SD.

To address the similarities and differences among inhibitory cytokines affecting humoral immunity, we first analyzed the effects of TGF-βs on B cells *in vitro*. Consistent with our previous report that TGF-β3 inhibited B cell proliferation under anti-IgM stimulation (3), TGF-β1 and TGF-β3 inhibited proliferation of B cells in the presence of anti-CD40 antibody-mediated co-stimulation without affecting cell death (Figure 1A; Figure S1A in Supplementary Material). Total IgG production under stimulation with anti-CD40 and IL-4 was also suppressed by TGF-β1 or TGF-β3 (Figure 1B). Further, either TGF-β1 or TGF-β3 suppressed the differentiation of B220^+^CD138^+^ plasmablasts in anti-CD40- and anti-IgM-stimulated B cells (Figure 1C). To examine their function *in vivo*, we constructed a plasmid pCAGGS vector (27, 28) that expressed *Tgfb1* or *Tgfb3*, thereby inducing high serum levels of these cytokines (Figures S1B in Supplementary Material) (3, 21, 27). Administration of pCAGGS-Tgfb3 inhibited the development of B220^+^GL-7^+^Fas^+^ GCB cells and suppressed antigen-specific antibody production in C57BL/6 mice immunized with a T cell-dependent antigen (Figures 1D,E; Figure S1C in Supplementary Material). In contrast, pCAGGS-Tgfb3 could not suppress CD4^+^CD25^−^CXCR5^+^PD-1^+^ T_FH_ cells with statistically significance (Figure S1D in Supplementary Material). On the other hand, pCAGGS-Tgfb1 could neither suppress NP-specific antibody production nor GCB cells (Figures 1D,E). These findings indicate that TGF-β3 could regulate T cell-dependent humoral immune responses *in vivo* by inhibiting B cells.

### Synergistic Inhibitory Effect of TGF-β and IL-10 on TLR-Related Humoral Immunity

**Figure 2:**
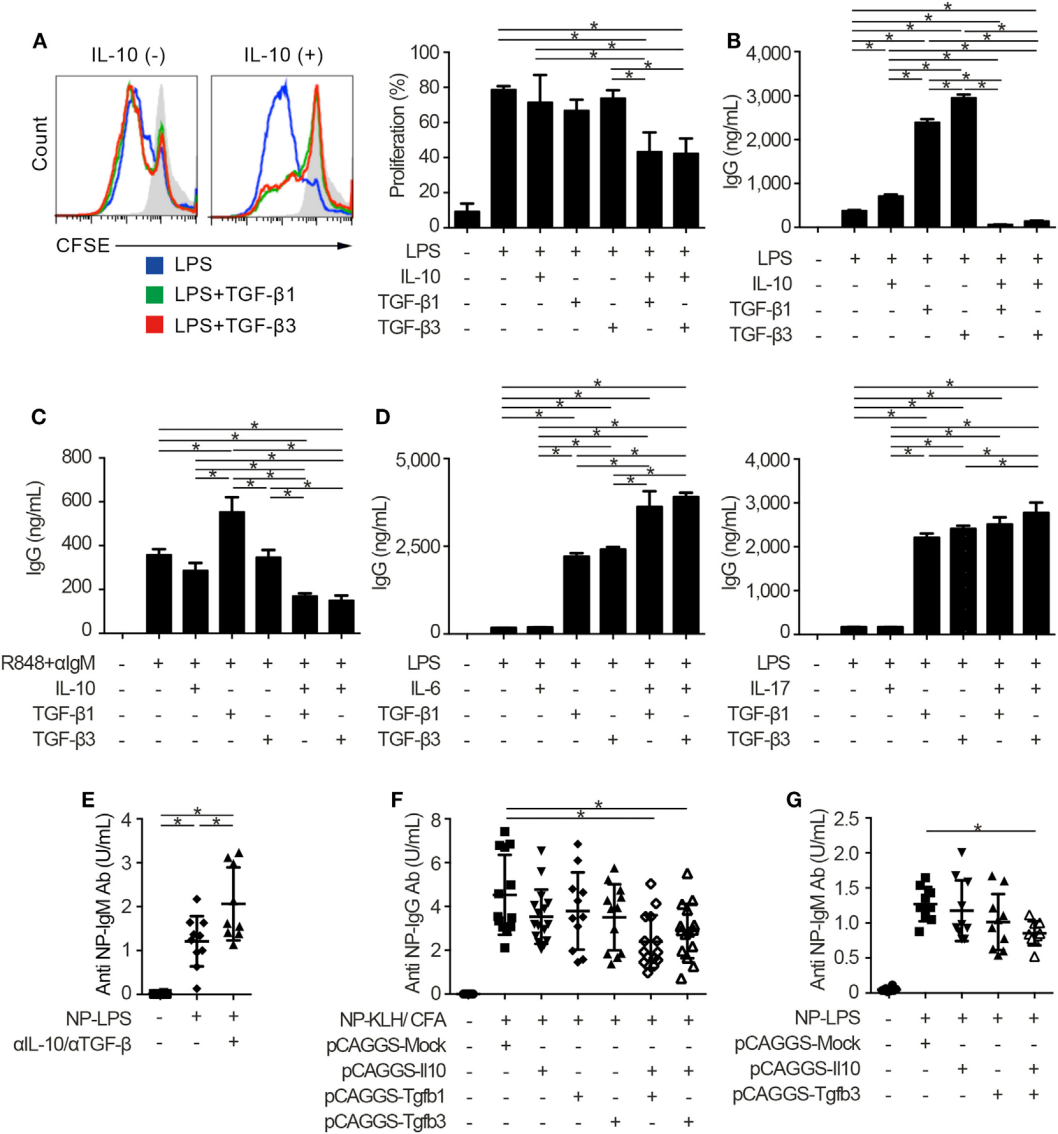
TGF-β and interleukin-10 (IL-10) synergistically suppress toll-like receptor-mediated humoral immune responses. **(A)** Histogram plots and percentages of CFSE-labeled B220^+^ B cells stimulated by 10 µg/ml lipopolysaccharides (LPS) either with or without 2 ng/ml TGF-β1, TGF-β3, and/or 50 ng/ml IL-10 for 3 days (*n* = 3). **(B)** Total IgG antibody titers in the supernatants of 3 µg/ml LPS-stimulated B cells either with 10 ng/ml TGF-β1, TGF-β3, and/or 50 ng/ml IL-10 for 7 days were quantified by ELISA (*n* = 3). **(C)** B cells were cultured under 200 ng/ml R848 and anti-IgM stimulation either with or without TGF-β1 or TGF-β3 for 7 days and total IgG antibody production was assessed by ELISA (*n* = 3). **(D)** Total IgG antibody titer in the supernatants of B cells cultured with LPS and 25 ng/ml IL-6 (left) or 1 ng/ml IL-17 (right) either with TGF-β1 or TGF-β3 for 7 days (*n* = 3). Data are representative of more than two independent experiments in **(A–D)**. **(E)** Serum anti-NP-bovine serum albumin (BSA) antibody titers from NP-LPS-immunized B6 mice treated with 300 µg anti-IL-10 antibody and 300 µg anti-TGF-β antibody were quantified by ELISA (*n* = 9–10). **(F,G)** Anti-NP-BSA antibody titers from NP-KLH/CFA-immunized (*n* = 11–16) **(F)** or NP-LPS-immunized (*n* = 9–10) **(G)** mice with indicated pCAGGS plasmid vectors were quantified. *P* < 0.05 by one-way analysis of variance followed by Bonferroni’s multiple comparisons test **(A–D)** or Holm–Sidak’s multiple comparisons test **(E–G)**. Error bars, SD.

Innate and adaptive immune systems cooperate in the pathogenesis of autoimmunity (29), and the characteristic expression of TLRs on B cells makes cell-intrinsic connections of innate signals to adaptive immunity (30, 31). Thus, we next examined whether TGF-βs could regulate TLR-stimulated B cell activation by using TLR stimulants which have pathogenic roles in SLE (14, 32). Notably, either TGF-β1 or TGF-β3 failed to inhibit LPS-stimulated B cell proliferation and instead enhanced the antibody production (Figures 2A,B; Figure S2A in Supplementary Material), consistent with previous observations (12, 33). It is well known that Tregs produce not only TGF-βs but also IL-10 (2–4, 6, 22). Then, we hypothesized that the concomitant existence of TGF-βs and IL-10 would be important for the regulation of humoral immune homeostasis. As expected, contrary to the single addition of TGF-β or IL-10, simultaneous addition of TGF-β and IL-10 inhibited the proliferation and antibody production of LPS-stimulated B cells (Figures 2A,B; Figure S2A in Supplementary Material). Similar results were observed in B cells stimulated with R848, which is a selective ligand for TLR7 (Figure 2C). We next verified whether TGF-β and cytokines other than IL-10 could show synergistic inhibitory effects on B cells. Although TGF-βs plus IL-6 provoke pro-inflammatory responses with inducing T helper 17 (Th17) cells as a cytokine synergy (34, 35), TGF-β plus IL-6 show a synergistic enhancement, but not a synergistic inhibition, in antibody production of B cells (Figure 2D). Also, a combination of TGF-β plus IL-17, a pro-inflammatory cytokine produced by Th17 cells (36), did not have synergistic inhibitory effects in antibody production of B cells (Figure 2D).

To assess immunological roles of TGF-β and IL-10 on TLR-related systemic immune responses *in vivo*, we investigated antigen-specific immune responses with blocking antibodies and pCAGGS plasmid vectors. Since both IL-10 and TGF-β have pro-inflammatory and inhibitory effects *in vivo* (12, 13), we firstly evaluated the effects of endogenously produced IL-10 and TGF-βs in T cell-independent TLR stimulation. As a result, the administration of anti-pan TGF-β and anti-IL-10 neutralizing antibodies enhanced the T-cell-independent immune responses (Figure 2E; Figures S2B,C in Supplementary Material).

Further, antigen-specific antibody production after the immunization with TLR-stimulating adjuvant (37) was suppressed with co-administration of pCAGGS-Tgfb1 or pCAGGS-Tgfb3 and pCAGGS-Il10, but not single pCAGGS plasmid vectors (Figure 2F). These observations suggest that the synergistic effects between IL-10 and either TGF-β1 or TGF-β3 are comparable. Although the therapeutic effects of TGF-β1 on SLE produced by CD4^+^CD25^+^Foxp3^+^ Tregs, which also produce IL-10 (2), have not been verified, IL-10-producing LAG3^+^ Tregs exist in the steady state and TGF-β3 produced by LAG3^+^ Tregs showed therapeutic effects on lupus pathologies (3, 6). Therefore, we focused on the cytokine synergy between TGF-β3 and IL-10, both of which are highly produced by LAG3^+^ Tregs (3, 6). To evaluate the synergistic inhibitory effects of TGF-β3 and IL-10 directly in mice models with innate immune signals, we analyzed 4-hydroxy-3-nitrophenylacetyl (NP)-LPS immunized mice treated with pCAGGS-Tgfb3 and/or pCAGGS-Il10. Intriguingly, simultaneous treatment with both pCAGGS-Tgfb3 and pCAGGS-Il10, but not with pCAGGS-Tgfb3 or pCAGGS-Il10 alone, effectively suppressed NP-LPS-induced antibody production (Figure 2G).

**Figure 3:**
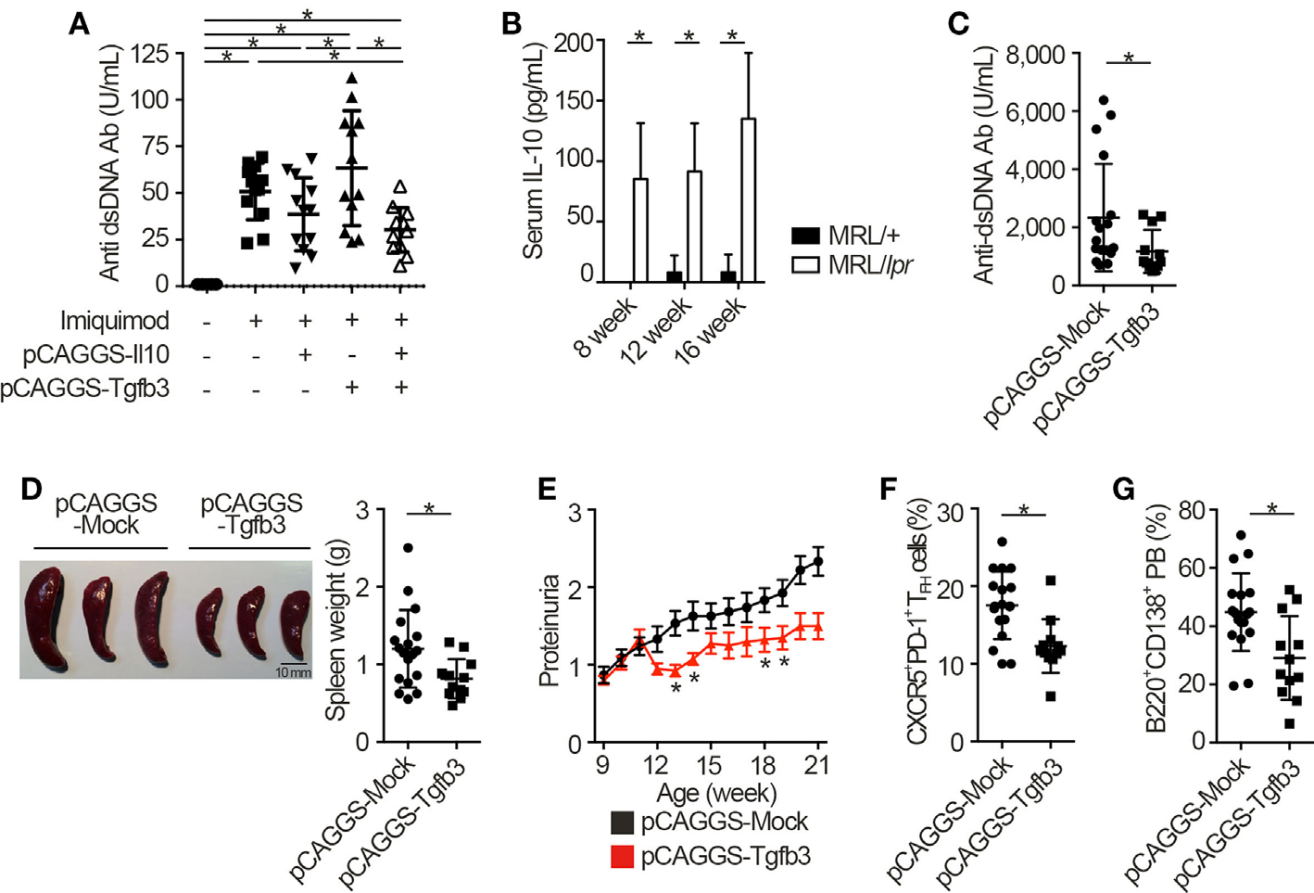
TGF-β3 exerts therapeutic effects in mouse models of lupus under the presence of interleukin-10 (IL-10). **(A)** Indicated pCAGGS vectors were i.v. administered every 2 weeks to BALB/c mice treated with 1.25 mg imiquimod epicutaneously three times weekly. Serum anti-dsDNA antibody titers were quantified by ELISA (*n* = 12). **(B)** Serum IL-10 levels in 8- to 16-week-old MRL/+ and MRL/*lpr* mice were analyzed by ELISA (*n* = 3). **(C–G)** MRL/*lpr* mice treated with indicated pCAGGS vectors every 4 weeks were analyzed at an age of 21 weeks. Serum anti-dsDNA antibody levels **(C)**, gross appearances and weights of spleens **(D)** (*n* = 12–18), and chronological proteinuria progression **(E)** (*n* = 12–25). Flow cytometric quantification of CXCR5^+^PD-1^+^ T_FH_ cells **(F)** and B220^+^CD138^+^ plasmablasts **(G)** (*n* = 12–18). *P* < 0.05 by one-way analysis of variance (ANOVA) followed by Holm–Sidak’s multiple comparisons test **(A)**, two-way ANOVA followed by Bonferroni’s multiple comparisons test **(B)**, Student’s *t*-test **(C,D,F,G)** or Mann–Whitney’s *U* test **(E)**. Error bars, SD. **(A–D,F,G)** and SEM. **(E)**.

Next, we injected each pCAGGS plasmid vectors to murine models of lupus in order to assess the therapeutic effects of these cytokines. Importantly, treatment with both pCAGGS-Tgfb3 and pCAGGS-Il10 suppressed autoantibody productions in a murine lupus model induced with TLR7 agonist, imiquimod (26) (Figure 3A; Figure S3A in Supplementary Material). TLR signaling is also implicated in the pathogenesis of lupus-prone MRL-*Fas^lpr/lpr^* (MRL/*lpr*) mice (38–40). On the other hand, the pathogenic role of IL-10 in systemic autoimmunity has been suggested previously. For example, elevated levels of serum IL-10 correlate with disease activity of SLE (41) and anti-IL-10 antibody exhibits therapeutic effects in SLE (42). Consistent with the previous report (43), we also confirmed elevated levels of serum IL-10 in MRL/*lpr* mice (Figure 3B). As expected, administration of pCAGGS-Tgfb3 alone showed therapeutic effects against anti-dsDNA antibody production, splenomegaly, and proteinuria progression in MRL/*lpr* mice (Figures 3C–E; Figure S3B in Supplementary Material) and suppressed the development of T_FH_ cells and B220^+^CD138^+^ plasmablasts (Figures 3F,G). On the contrary, the MRL/*lpr* mice treated with pCAGGS-Tgfb3 did not exhibit a decrease in the number of CD4^+^ T cells and B220^+^ B cells (Figures S3C,D in Supplementary Material), although B cells and T cells play central roles in the pathogenesis of lupus phenotypes in MRL/*lpr* mice (44–46). These results indicate that TGF-β and IL-10 synergistically inhibit TLR-related humoral immune responses, and TGF-β3 has therapeutic potentials for the lupus-prone mice.

### Suppression of Mammalian Target of Rapamycin Complex 1 (mTORC1)-Directed Signaling in B Cells by TGF-β3 and IL-10

**Figure 4:**
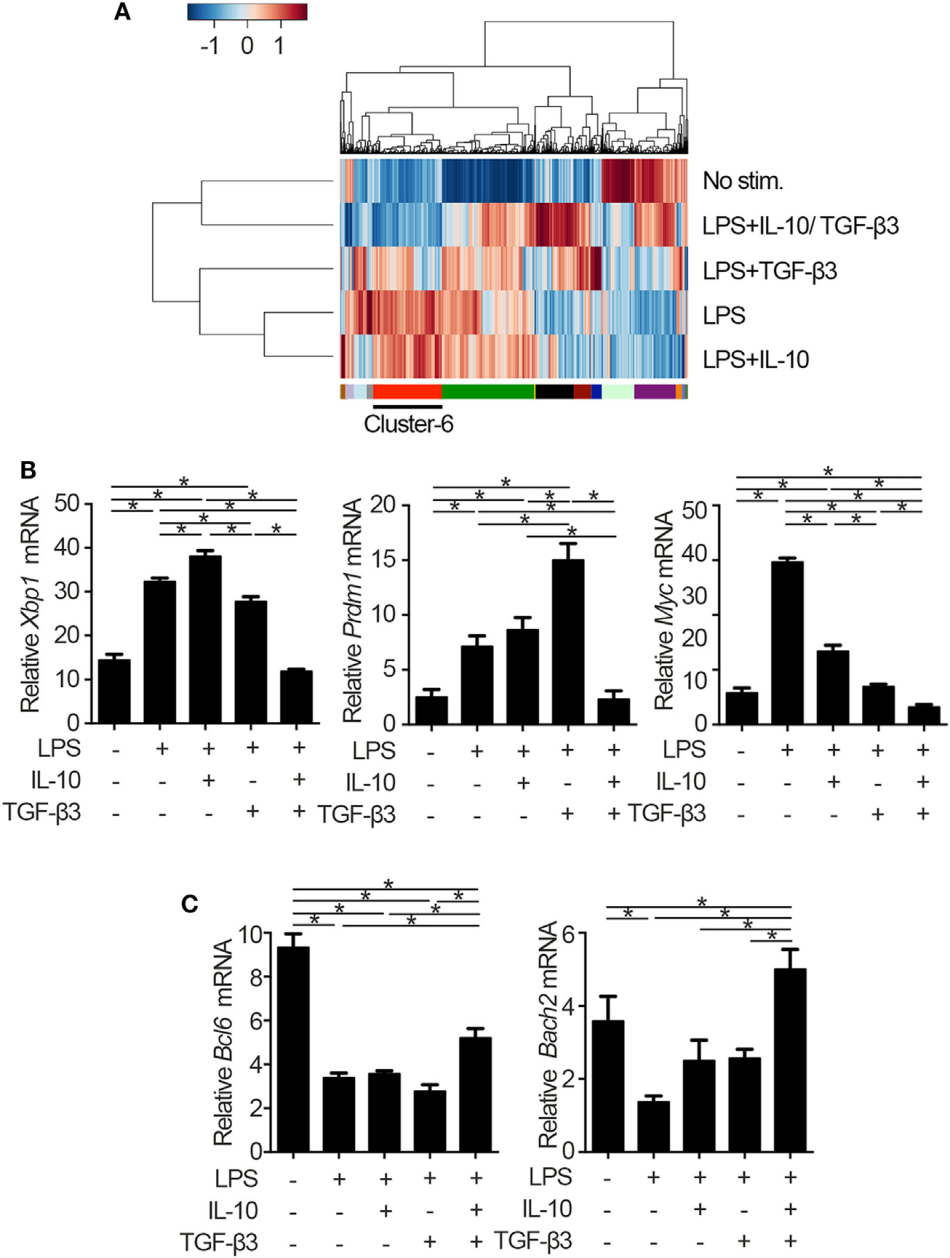
Cytokine synergy of TGF-β3 and interleukin-10 (IL-10) modulate transcriptional programs in lipopolysaccharide (LPS)-stimulated B cells. **(A)** Heatmap visualization and hierarchical clustering analysis of differentially expressed genes of whole genome RNA-sequencing from B220^+^7-AAD^−^ B cells stimulated by LPS with either TGF-β3 and/or IL-10 for 3 days. The clustered genes were subdivided into 16 categories labeled by different colors. **(B,C)** qRT-PCR analysis of genes downregulated **(B)** or upregulated **(C)** with TGF-β3 and IL-10 more than those in LPS-stimulated B cells (*n* = 3). *P* < 0.05 by one-way analysis of variance followed by Bonferroni’s multiple comparisons test **(B,C)**. Error bars, SD.

**Figure 5:**
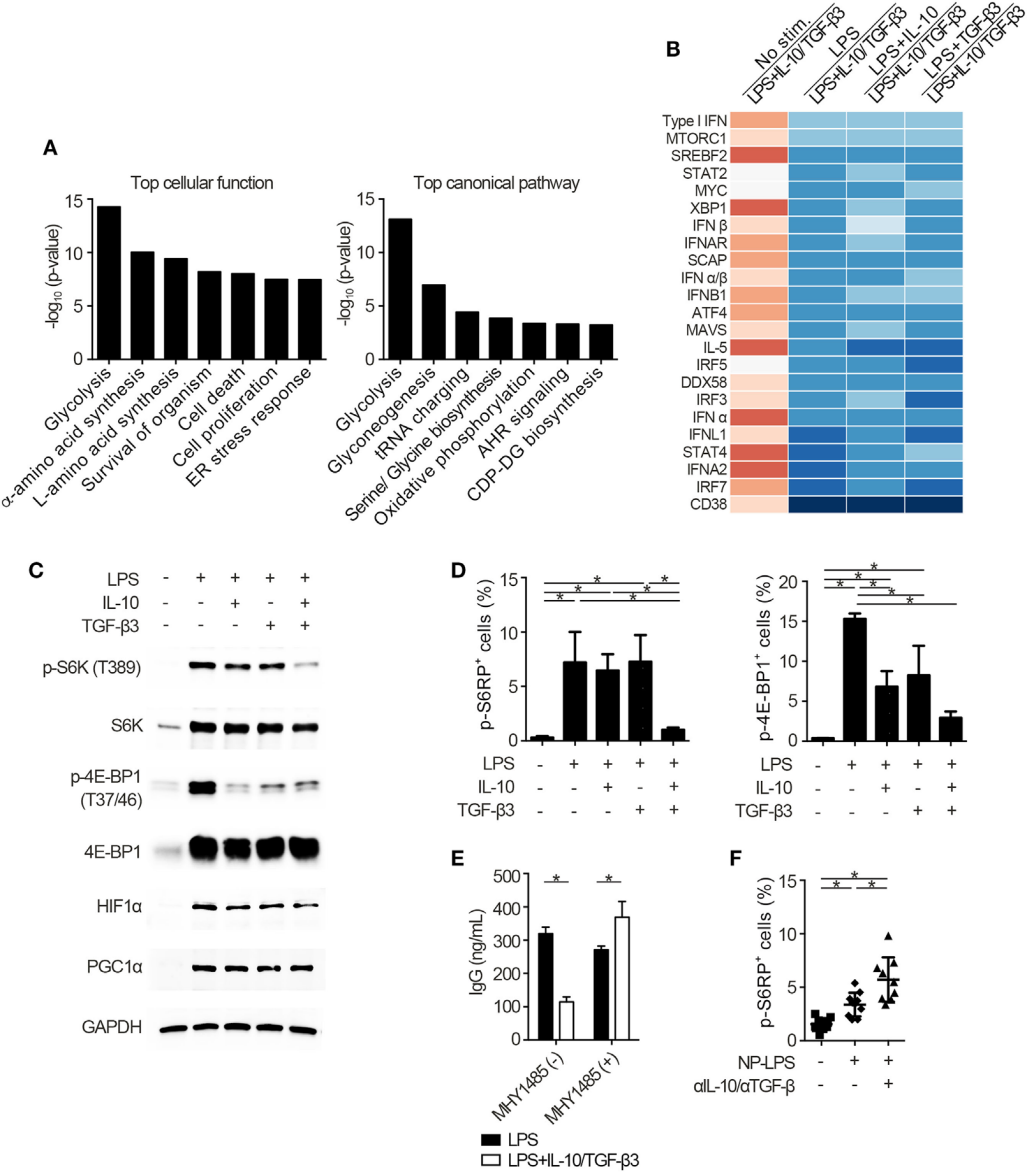
Cytokine synergy of TGF-β3 and interleukin-10 (IL-10) inhibits mammalian target of rapamycin complex 1 (mTORC1) activity in lipopolysaccharide (LPS)-stimulated B cells. **(A)** The seven most related cellular functions and canonical pathways in genes of cluster 6 in Figure 4A were analyzed by in Ingenuity Pathway Analysis (IPA) software. **(B)** Heatmap visualization of activation *z*-score ratios of LPS-stimulated B cells to LPS-stimulated B cells with IL-10/TGF-β3 less than −3.1 calculated by the upstream regulator analysis in IPA software. All of the 1,895 differentially expressed genes depicted in Figure 4A were utilized in this analysis. **(C)** Representative western blot analyses of total and phosphorylated protein levels in LPS-stimulated B cells treated either with TGF-β3 and/or IL-10 for 24–48 h. **(D)** Flow cytometric (FCM) quantification of phosphorylated S6RP at Ser235/236 and 4E-BP1 at Thr37/46 in LPS-stimulated B cells either with or without TGF-β3 and/or IL-10 for 72 h (*n* = 3). **(E)** Total IgG antibody titers in the supernatants of LPS-stimulated B cells with or without TGF-β3 and IL-10 in the presence or absence of 2 µM MHY1485 for 7 days quantified by ELISA (*n* = 3). Data are representative of more than two independent experiments in **(C–E)**. **(F)** FCM quantification of phosphorylated S6RP at Ser235/236 in splenic B220^+^ cells from B6 mice treated as in Figure 2E (*n* = 9–10). *P* < 0.05 by one-way **(D,F)** or two-way **(E)** analysis of variance followed by Bonferroni’s multiple comparisons test. Error bars, SD.

To identify the mechanisms underlying the inhibitory cytokine synergy, we conducted RNA-sequencing of LPS-stimulated B cells cultured in the presence of TGF-β and/or IL-10. Gene clustering of DEGs categorized the genes into 16 clusters; genes in cluster 6 showed a characteristic gene expression pattern that was enhanced by either TGF-β3 or IL-10 alone and suppressed by TGF-β3 and IL-10 in combination (Figure 4A). Quantitative PCR analyses confirmed that genes related to cell proliferation, plasma cell differentiation, and endoplasmic reticulum-stress responses, such as *Myc, Prdm1*, and *Xbp1*, were suppressed, although transcriptional repressors such as *Bcl6* and *Bach2* were upregulated, in LPS-stimulated B cells treated with TGF-β3 and IL-10 (Figures 4B,C; Figure S4A in Supplementary Material). Intriguingly, simultaneous phosphorylation of STAT3 and Smad3 was observed in LPS-stimulated B cells with IL-10 and TGF-β3 (Figures S4B,C in Supplementary Material). This finding might suggest the importance of a cross talk with STAT3 and Smad3 for the cooperative action of the two cytokines. We next examined specific transcriptional regulation observed in TGF-β3- and IL-10-treated B cells. Pathway analyses of genes in the cluster 6 in Figure 4A suggested involvement of energy metabolism in TGF-β3- and IL-10-treated B cells (Figure 5A). Potential upstream regulators predicted by Ingenuity pathway analysis (IPA) software showed that TGF-β3 and IL-10 downregulated mTORC1 signaling, which plays a central role in cellular metabolism (47, 48) (Figure 5B). mTOR signaling is important in enhancement of humoral immune responses (49), and an mTORC1 inhibitor ameliorates lupus pathologies (50). Thus, we evaluated the phosphorylation of downstream targets of mTORC1. Although the phosphorylation of eukaryotic translation initiation factor 4E-binding protein 1 (4E-BP1) was inhibited by TGF-β3 or IL-10 alone (Figures 5C,D; Figure S4D in Supplementary Material), the combination of TGF-β3 and IL-10 only inhibited phosphorylation of both 4E-BP and S6 kinase (S6K) (Figures 5C,D; Figure S4D in Supplementary Material). Expression of hypoxia inducible factor-1α (HIF1α) which regulates glycolysis (51, 52) tended to decrease under the combination of TGF-β3 and IL-10. As expected, mTORC1 activator MHY1485 (53) counteracted the synergistic inhibitory effect of TGF-β3 and IL-10 on antibody production (Figure 5E). Further, we administered anti-IL-10 and anti-TGF-β neutralizing antibody to NP-LPS-immunized mice and analyzed intracellular phosphorylation level of S6 in splenic B cells. Blockade of IL-10 and TGF-β induce phosphorylation of S6 in B cells (Figure 5F; Figure S4E in Supplementary Material). These findings indicate that TGF-β3 and IL-10 synergistically regulate genetic programs through modulating mTORC1 signaling in B cells activated by innate immunity signals.

### Suppression of TGF-β3-Induced Upregulation of Autophagy by IL-10 in LPS-Stimulated B Cells

**Figure 6:**
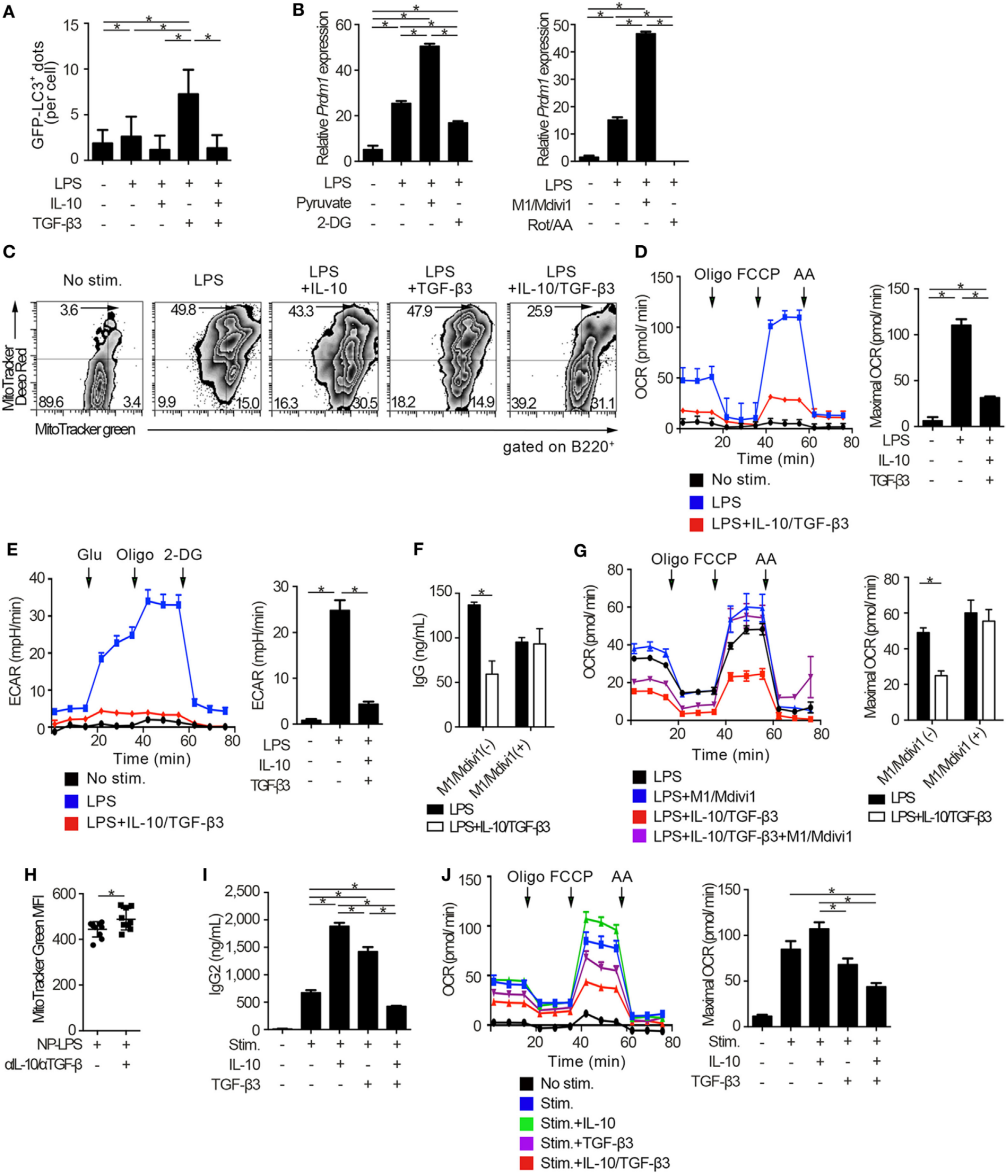
Cytokine synergy of TGF-β3 and interleukin-10 (IL-10) regulates metabolism in toll-like receptor-stimulated B cells. **(A)** Splenic B cells from LC3-green fluorescent protein (GFP) mice were stimulated by lipopolysaccharides (LPS) either with TGF-β3 and/or IL-10 for 3 days. GFP^−^LC3^+^ spots captured by fluorescence microscopy were counted manually (*n* = 20). **(B)** Relative Prdm1 expression in LPS-stimulated B cells either with 1 mM 2-DG, 10 mM sodium pyruvate, 0.5 µM rotenone (Rot)/antimycin A (AA), or 10 µM M1/Mdivi1 analyzed by qRT-PCR (*n* = 3). **(C)** Flow cytometric analysis of MitoTracker-stained, LPS-stimulated B cells with each cytokine cultured for 3 days. **(D,E)** Oxygen consumption rate (OCR) **(D)** and ECAR **(E)** of LPS-stimulated B cells with each cytokine measured by extracellular flux analyzer (*n* = 3). **(F,G)** IgG antibody titers quantified by ELISA **(F)** and OCR measured by extracellular analyzer **(G)** of LPS-stimulated B cells with or without TGF-β3 and IL-10 in the presence or absence of 10 µM M1/Mdivi1 (*n* = 3). **(H)** Mean fluorescence intensity (MFI) of MitoTracker Green in splenic B220^+^ cells from B6 mice treated as in Figure 2E (*n* = 9–10). **(I)** Human B cells were stimulated by 2.5 µg/ml CpG-ODN, 1,000 U/ml IL-2, 10 ng/ml IL-6, and 0.5 µg/ml anti-CD40 with 10 pg/ml TGF-β3, 10 ng/ml IL-10, and/or 1 µg/ml anti-IL-10 Ab for 7 days (*n* = 3). IgG2 antibody titers of the supernatants were quantified by ELISA. **(J)** OCR of CpG-ODN-stimulated human B cells with each cytokine for 3 days measured by extracellular flux analyzer (*n* = 3). *P* < 0.05 by one-way **(A,B,D,E,I,J)**, two-way **(F,G)** analysis of variance followed by Bonferroni’s multiple comparisons test, or Student’s *t*-test **(H)**. Error bars, SD. **(B,F,H,I)** and SEM. **(D,E,G,J)**.

We went on to examine whether autophagic activities could be modulated through the suppression of mTORC1 signaling by the combination of TGF-β3 and IL-10. Autophagy is a conserved self-degradative process (54) essential for plasma cell development (55). Although mTORC1 signaling negatively regulates autophagy (56), enhanced mTORC1 signaling in LPS-stimulated B cells is essential for cell proliferation (57) and also high autophagic activities are observed in those cells (55). To determine autophagic activities *in vivo*, we utilized mice expressing transgenic GFP fused to microtubule-associated protein 1A/1B-light chain 3 (LC3) (25). As expected, LPS stimulation on B cells increased formation of GFP-LC3^+^ dots and GFP fluorescence, and TGF-β3 further enhanced the GFP-LC3^+^ dots and GFP fluorescence (Figure 6A; Figures S5A,B in Supplementary Material). TGF-β3 prominently enhanced the GFP-LC3^+^ dots in LPS-stimulated B cells (Figure 6A; Figure S5A in Supplementary Material), and also enhanced GFP fluorescence analyzed by flow cytometry (Figure S5B in Supplementary Material). Although IL-10 alone did not affect the autophagic activity in LPS-stimulated B cells, IL-10 counteracted the enhancement of autophagosome formation mediated by TGF-β3 (Figures S5A,B in Supplementary Material). Next, we assessed whether the increased autophagic activities with TGF-β3 could affect antibody production of LPS-stimulated B cells by using 3-methylademine (3-MA), an autophagy inhibitor. As expected, the enhanced antibody production by TGF-β3 in LPS-stimulated B cells was suppressed with 3-MA (Figure S5C in Supplementary Material), autophagic activities might be partially involved in TGF-β3-induced enhancement of antibody production. Taken together, the simultaneous presence of IL-10 with TGF-β3 might be necessary for suppression of the TGF-β3-induced upregulation of autophagy.

### TGF-β3 and IL-10-Mediated Inhibition of B Cell Metabolic Capacity

There is growing interest in bioenergetic homeostasis as a target of immunotherapy (50, 52, 58). It was reported that activated B cells utilize both glycolysis and oxidative phosphorylation (59). To evaluate the direct effects of metabolic modulators, we utilized pyruvate, which is a substrate of mitochondrial respiration in plasma cells (60) and a combination of mitochondrial fusion promoter M1 and fission inhibitor Mdivi1, which enhances mitochondrial respiration (61). As expected, pyruvate or a combination of M1 and Mdivi1 upregulated *Prdm1*, a key transcriptional regulator for the generation of antibody-secreting cells (62), in LPS-stimulated B cells. In contrast, glycolysis inhibitor, 2-deoxyglucose (2-DG), or a combination of mitochondrial inhibitors, rotenone and antimycin-A (Rot/AA), suppressed *Prdm1* expression (Figure 6B). In accordance with these observations, TGF-β3 and IL-10 synergistically regulated genetic programs related to energy metabolism, such as *Cox5a, Cox5b, Ndufs7*, and *Atp5d* expression (Figure S6A in Supplementary Material). Then we hypothesized that B cell energy metabolism could be suppressed by TGF-β3 and IL-10 with relevance to the inhibition of mTORC1 signaling, and we used a combination of membrane potential independent stain MitoTracker Green and membrane potential dependent stain MitoTracker DeepRed to assess respiring mitochondria (63, 64). As expected, the combination of TGF-β3 and IL-10, but not single addition of TGF-β3 or IL-10, suppressed mitochondrial membrane potential in LPS-stimulated B cells (Figure 6C). Further, extracellular flux analyses revealed that both glycolysis and oxidative phosphorylation, which were enhanced by LPS stimulation, showed significant reduction in the presence of both TGF-β3 and IL-10 (Figures 6D,E). Importantly, M1/Mdivi1 counteracted the inhibitory effect of TGF-β3 and IL-10 through enhancing maximal OCR (Figures 6F,G). Further, *in vivo* combination of anti-TGF-β and anti-IL-10 neutralizing antibodies enhanced the mitochondrial membrane potential in B cells from NP-LPS-immunized mice (Figure 6H). These results indicate that the synergistic inhibition by TGF-β3 and IL-10 on energy metabolism determines B cell immune function.

To further investigate whether these findings observed in mice could hold true in humans, we analyzed the effects of TGF-β3 and IL-10 on TLR-stimulated B cells isolated from healthy humans. We recently reported that high concentrations of TGF-β3 (1 ng/ml) alone could effectively suppress CpG-oligodeoxynucleotides (ODN)-stimulated human B cell antibody production with inhibition of BLIMP-1 expression (65). Nonetheless, determining the role of TGF-β3 at physiological concentration is of interest for therapeutic purposes in humans. In healthy humans, the median serum concentrations of TGF-β3 is at the 11.8 pg/ml (66). As expected, neither IL-10 nor 10 pg/ml TGF-β3 alone inhibited metabolic signals, but addition of IL-10 with 10 pg/ml TGF-β3 effectively suppressed CpG-ODN-stimulated human B cell antibody production with reduced oxidative phosphorylation and glycolysis (Figures 6I,J; Figures S6B,C in Supplementary Material). These results collectively indicated that the inhibitory cytokine synergy of TGF-β3 and IL-10 on TLR-stimulated B cells occurs *via* suppression of energy metabolism in a physiological condition and is a key mechanism for induction of humoral immune tolerance.

## Discussion

TGF-βs and IL-10, potent inhibitory cytokines, have been investigated for their therapeutic potential in systemic inflammatory diseases (12, 13). However, the immunostimulatory effects of IL-10 on B cells and humoral immunity including lupus pathologies (12), and pleiotropic immunological roles of TGF-βs (13) make application of these potent inhibitory cytokines elusive. Herein, we proposed an inhibitory cytokine synergy with TGF-β and IL-10 that control TLR-related humoral responses via controlling glycolysis and oxidative phosphorylation of B cells. Although recent evidence indicates a role for IL-10 in SLE pathogenesis (41, 42), it was also reported that lupus like phenotypes of TGF-β3-sufficient MRL/*lpr* mice are exacerbated by genetic deletion of IL-10 (67). These findings suggest that physiological expression levels of IL-10 might be necessary for the control of lupus pathology. Although the mechanisms of action of these cytokines in immune cells remained poorly elucidated, latest reports uncovered that TGF-β signaling inhibited natural killer cell activation *via* suppressing mTOR signaling (68) and IL-10 inhibited LPS-induced macrophage activation *via* suppressing glycolysis (63). There is growing evidence that metabolic reprogramming affects immune function in macrophages, and LPS-stimulated pro-inflammatory M1 macrophages mainly rely on aerobic glycolysis, while IL-4-mediated anti-inflammatory M2 macrophages enhance oxidative phosphorylation and fatty acid oxidation (69). In B cells, mTORC1 activity is recognized to be important for the differentiation and antibody production, and metabolic reprogramming upon various stimulation determines the fate of B cells (70). Controlling mTORC1 and glycolysis by inhibitory cytokines is predicted to regulate humoral immunity (70) but cytokine-mediated metabolic inhibition in B cells has not been reported. Our results showed that a combination of TGF-β and IL-10 inhibited TLR-stimulated immune reaction with suppressing bioenergetics of B cells, and an mTORC1 activator or a reagent enhancing mitochondrial respiration counteracted the synergistic inhibitory effects. TLR stimulation is supposed to be essential in the activation of autoreactive B cells (31). Accumulating evidences has highlighted the central role of TLR signaling in the induction of immune responses in SLE (19). Thus, the inhibitory cytokine synergy that regulate the TLR signaling-mediated metabolic signals in B cells have a potential to be a novel therapeutic strategy for autoantibody-related autoimmune diseases.

Patterns of cytokine combination determine its distinct synergistic effects on the target cells (24). In T cells, TGF-β is required signal for Treg differentiation, but co-existence of TGF-β plus IL-6 and IL-23 induce Th17 differentiation (35). Although a combination of TGF-β and pro-inflammatory cytokines, such as IL-12 and IL-23, could induce a specific transcriptional factors and downstream signaling (24, 71), synergistic inhibition by a combination of inhibitory cytokines has not been previously elucidated. Here, we uncovered TGF-β3 and IL-10-mediated synergistic inhibition of TLR-stimulated B cells *via* modulating energy metabolisms. We showed cooperative transcriptional modulation including suppressing mTOR signaling by a combination of TGF-β and IL-10 in TLR-stimulated B cells through RNA-sequencing. Although the molecular mechanisms of cytokine synergy have not fully elucidated, synergy through cooperative action is supposed to occur at the transcriptional level (24). Previous reports indicate that that Smad-STAT signaling networks are cell-type specific and context-dependent. For example, a direct cross talk between Smad3 and STAT3 is bridged by p300 in hepatoma cells (72), a synergistic action of LIF-induced-Smad1 and BMP2-induced-STAT3 is bridged by p300 in neural cells (73), and Smad2/3 and STAT3 cooperatively interact in Th17 cell differentiation (74). In this study, we observed simultaneous activation of STAT3 and Smad3 in LPS-stimulated B cells with IL-10 and TGF-β3. Further studies should be conducted to verify the cross talk with STAT3 and Smad3 mediated by the inhibitory cytokine synergy in LPS-stimulated B cells.

B cell-specific mTOR deficiencies are associated with decreased high-affinity antibody production (49), and enforced mTOR signaling promotes plasma cell differentiation and immunoglobulin production (75). Enhanced glycolytic states in activated B cells (50, 59) and requirement of mitochondrial pyruvate for long-lived plasma cells (60) imply the importance of inhibition of mTORC1 signaling, glycolysis, and mitochondrial respiration in B cell lineage to regulate excessive humoral immune responses. Biological processes in immune cells are supported by energy metabolism, and immune-metabolism relationships could be a basis for future therapy (52, 76). Indeed therapeutic effects of metabolic intervention were observed in models of SLE and transplantation, and rapamycin, an mTORC1 inhibitor, and pioglitazone, a selective peroxisome proliferator-activated receptor-γ agonist, is currently being tested in a clinical trial for SLE (50, 58, 76). The fact that CD4^+^CD25^+^ Tregs produce both TGF-β1 and IL-10 (2, 4), and CD4^+^CD25^−^LAG3^+^ Tregs produce both TGF-β3 and IL-10 (3, 6, 22) imply the existence of a mechanism for maintaining humoral immune tolerance by the co-production of these cytokines. In this study, although both of TGF-β1 and TGF-β3 effectively suppressed B cell activation *in vitro*, only TGF-β3 effectively regulated the humoral immune responses *in vivo*. It is well known that extracellular activation of latent TGF-β are mediated by proteases, oxygen radicals, thrombospondin type I, integrins, glycoprotein A repetitions predominant, etc. (77). The difference of therapeutic effects between pCAGGS-Tgfb3 and pCAGGS-Tgfb1 *in vivo* might be due to their complexed physiological activation mechanisms including multiple factors of extracellular matrix components. Further, we showed that TGF-β3 ameliorated lupus pathologies with suppressing T_FH_ cells and antibody-producing cells while maintaining total CD4^+^ T cells and B220^+^ B cell count in IL-10-sufficient MRL/*lpr* mice. The cell-type selective effects of TGF-β3 for targeting GC responses could have an advantage in adverse effects such as opportunistic infection typically caused by other immunosuppressive therapies. Potent anti-fibrotic properties of TGF-β3 (78), while TGF-β1 has strong pro-fibrotic effects (13) further ensure the safety for the therapeutic application.

In summary, our findings indicate that the combined presence of TGF-β3 and IL-10 in a local environment could target humoral immune responses. Regulation mediated by inhibitory cytokine synergy could be a future therapeutic option for autoimmune diseases, including SLE.

## Ethics Statement

This study was carried out in accordance with the guidelines of the ethics committee of the University of Tokyo Institutional Animal Care and Use Committee. The protocol was approved by the ethics committee of the University of Tokyo Institutional Animal Care and Use Committee (approval number: G10095). Also, this study was carried out in accordance with the guidelines of the ethics committee of the University of Tokyo. The protocol was approved by the ethics committee of the University of Tokyo (approval number: G3582). All subjects gave written informed consent in accordance with the Declaration of Helsinki.

## Author Contributions

All authors extensively contributed to the work presented in this paper. TK and MI carried out all experiments and contributed equally to this work. TK, MI, TO, KM, YI, SS, HS, KY, and KF conceived, designed, and analyzed the experiments and contributed to writing the manuscript.

## Conflict of Interest Statement

TO received financial support or fees from Chugai and Bristol-Myers Squibb (BMS). KY received financial support or fees from AbbVie, Astellas, BMS, Daiichi-Sankyo, Mitsubishi Tanabe, Pfizer, Sanofi, Santen, Takeda, Teijin, Boehringer Ingelheim, Chugai, Eisai, Ono, Taisho Toyama, UCB, ImmunoFuture, Asahi Kasei, Janssen, and NIPPON KAYAKU. KF received financial support or fees from Astellas, BMS, Daiichi-Sankyo, Mitsubishi Tanabe, Pfizer, Ayumi, Takeda, Chugai, Eisai, Taisho Toyama, UCB, Janssen, Eli Lilly, and NIPPON KAYAKU. The remaining authors declare no competing financial interests. TK, KM, TO, KY, and KF received patent-licensing arrangements with Chugai.
