# Supplementary material for: Transforming Growth Factor-β and Interleukin-10 Synergistically Regulate Humoral Immunity via Modulating Metabolic Signals
Source: Front Immunol. 2018 Jun 14;9:1364. doi: 10.3389/fimmu.2018.01364 (PMC6010538; doi:10.3389/fimmu.2018.01364)
Supplement: Supplementary file 1 [file data_sheet_1.DOCX]

Supplementary Material

Transforming growth factor-β and interleukin-10 synergistically regulate humoral immunity via modulating metabolic signals

Toshihiko Komai^1,5^, Mariko Inoue^1,5^, Tomohisa Okamura^1,2,3*^, Kaoru Morita^1^, Yukiko Iwasaki^1^, Shuji Sumitomo^1^, Hirofumi Shoda^1^, Kazuhiko Yamamoto^1,3,4^, and Keishi Fujio^1*^

^1^ Department of Allergy and Rheumatology, Graduate School of Medicine, The University of Tokyo, Tokyo, Japan

^2^Department of Functional Genomics and Immunological Diseases, Graduate School of Medicine, The University of Tokyo, Japan

^3^ Max Planck-The University of Tokyo Center for Integrative Inflammology, The University of Tokyo, Tokyo, Japan

^4^ Laboratory for Autoimmune Diseases, Center for Integrative Medical Sciences, RIKEN, Kanagawa, Japan

^5^ These authors contributed equally

*** Correspondence:**Tomohisa Okamura, and Keishi Fujio
tomohisa-tky@umin.ac.jp (TO), and kfujio-tky@umin.ac.jp (KF)


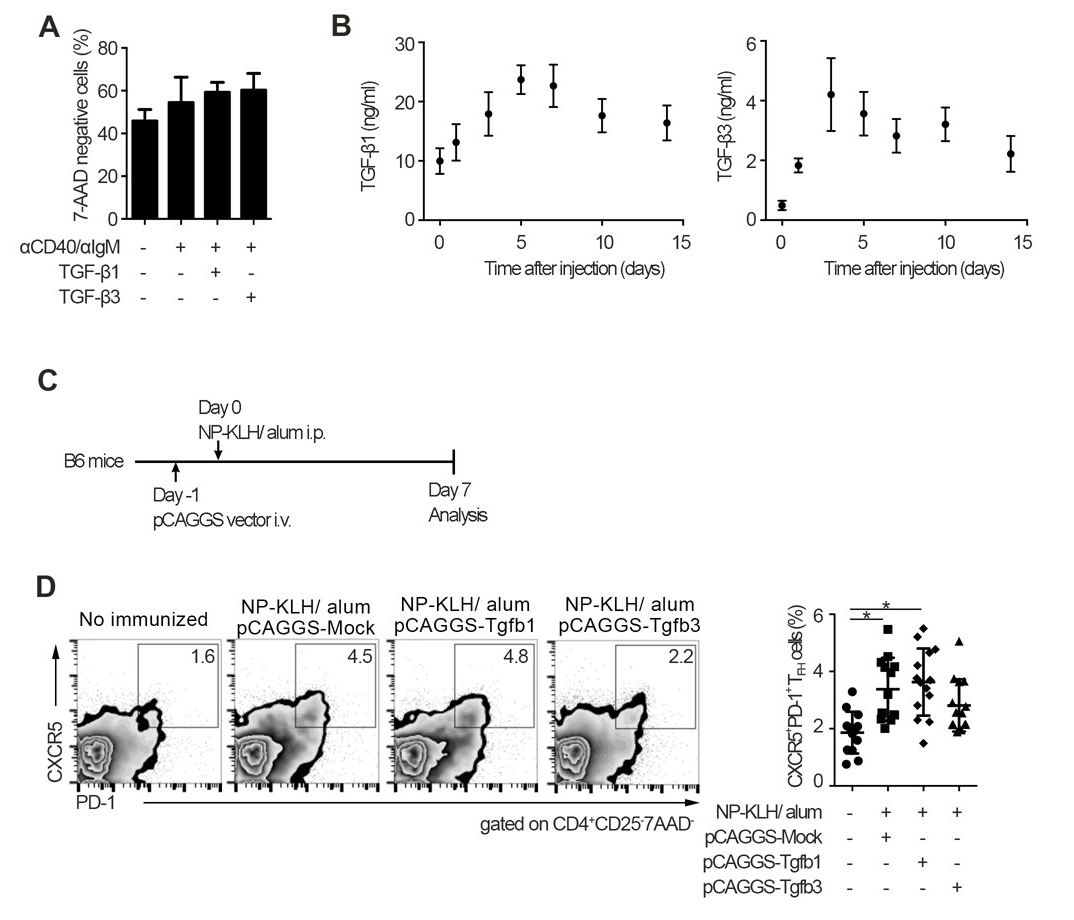


**FIGURE S1.** **TGF-β regulates T cell-dependent humoral immune responses by inhibiting B cells. (A)** Percentages of 7-AAD negative live cells in cultured B cells stimulated with anti-CD40 and anti-IgM in the presence of TGF-β1 or TGF-β3 for 3 days (*n* = 3). **(B)** Plasma TGF-β1 levels (left) and serum TGF-β3 levels (right) of C57BL/6 (B6) mice after intravenous injection of 100 μg pCAGGS-Tgfb1 or pCAGGS-Tgfb3 plasmid vectors (*n* = 6). Concentration of TGF-β1 and TGF-β3 were quantified at the indicated time points after the injection by ELISA. **(C)** Experimental scheme for the analysis of the effects of TGF-β under immunization with T cell-dependent antigen. B6 mice treated with pCAGGS plasmid vectors were immunized by i.p. injection of 100 μg NP-KLH in alum. **(D)** Flow cytometric (FCM) plots and quantification of CXCR5^+^PD-1^+^ T_FH_ cells in CD4^+^CD25^-^7AAD^-^ cells from B6 mice administered the indicated pCAGGS vectors and immunized with 100 μg NP-KLH in alum (n = 13-14). *P* < 0.05 by one-way ANOVA followed by Holm-Sidak’s multiple comparisons test. Error bars, s.e.m. (B) and s.d. (A, D).

**
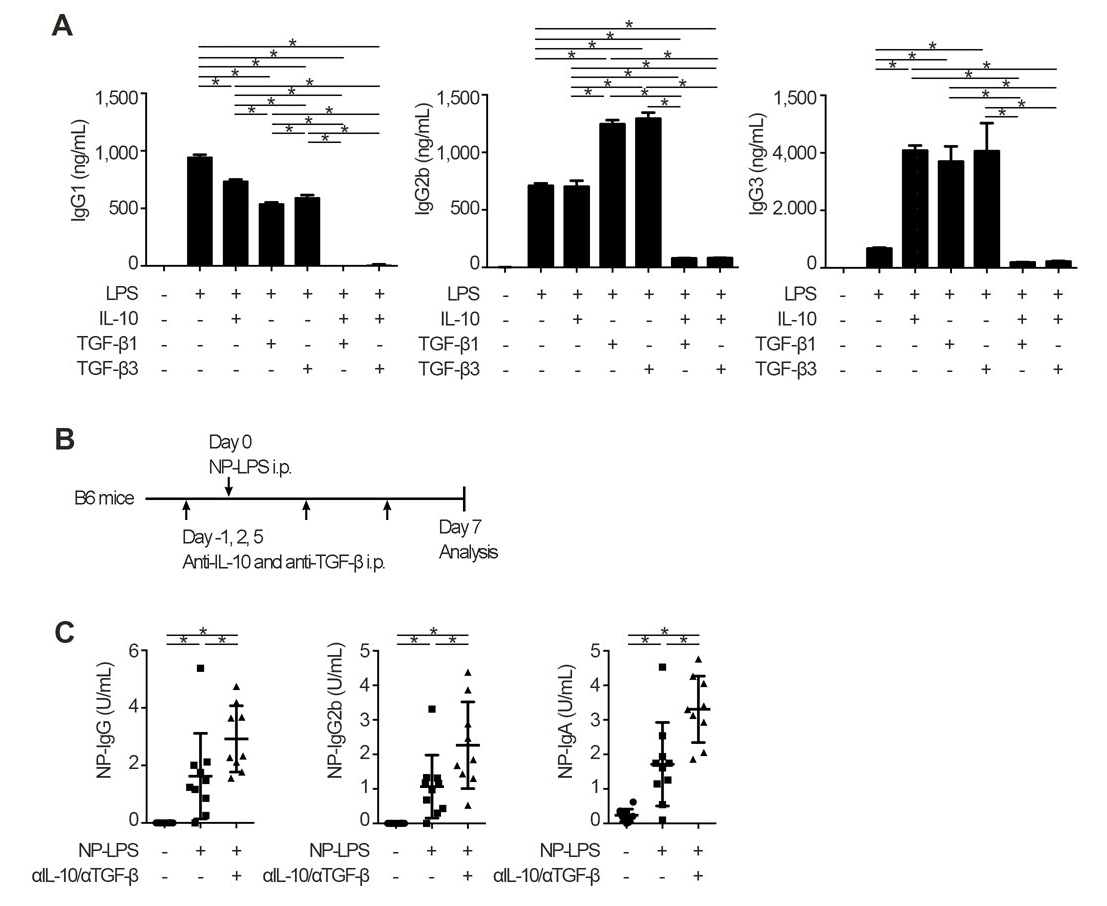
**

**FIGURE S2. TGF-β and IL-10 synergistically suppress TLR-related B cell activation. (A)** IgG1, IgG2b, and IgG3 antibody titers in the supernatants of LPS-stimulated B cells either with or without TGF-β1 or TGF-β3 and IL-10 for 7 days quantified by ELISA (*n* = 3). **(B)** Experimental scheme for the analysis of the effects of anti-IL-10 and anti-TGF-β neutralizing antibodies under immunization with T cell-independent antigen. B6 mice were immunized with NP-LPS on day 0 and 300 μg anti-IL-10 antibody and 300 μg anti-TGF-β antibody were administered on days -1, 2, and 5. **(C)** Antibody titers against NP-IgG, NP-IgG2b and NP-IgA were determined by ELISA (n = 9-10). *P* < 0.05 by one-way ANOVA followed by Bonferroni’s multiple comparisons test. Error bars, s.d..


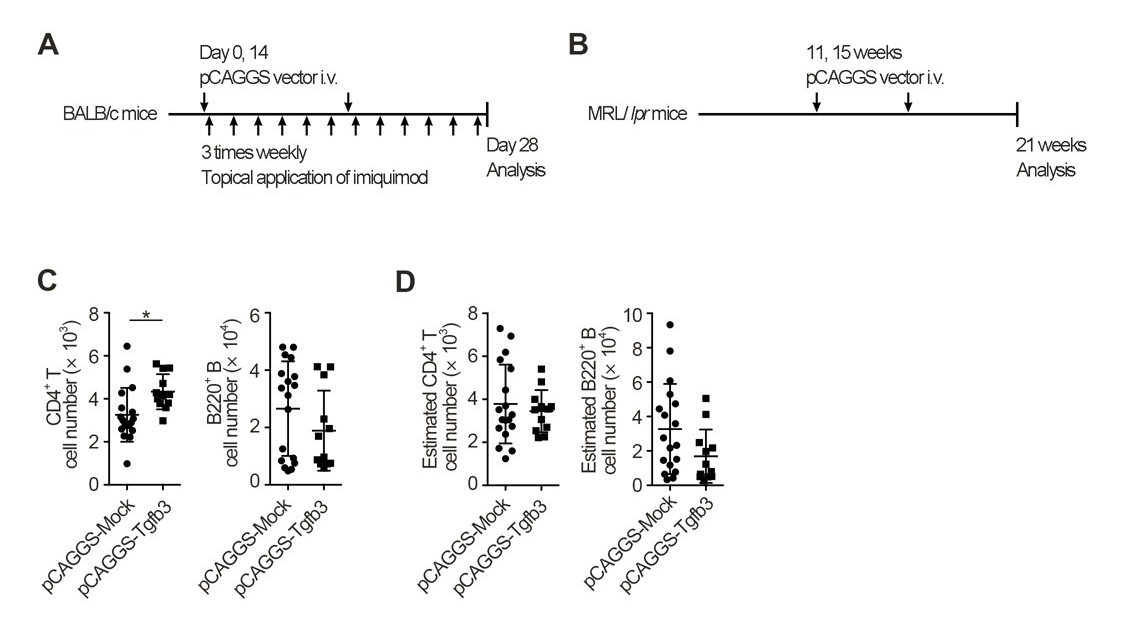


**FIGURE S3. The effect of TGF-β3 in imiquimod-treated or MRL/*lpr* lupus prone mice. (A)** Experimental scheme for the analysis of the imiquimod-induced lupus model mice treated with pCAGGS-Tgfb3 and pCAGGS-Il10 vectors. pCAGGS vectors were administered i.v. every 2 weeks to BALB/c mice treated with 1.25 mg imiquimod epicutaneously 3 times weekly. **(B)** Experimental scheme for the analysis of the MLR/*lpr* mice treated with pCAGGS-Tgfb3 vector. MRL/*lpr* mice were treated with indicated pCAGGS vectors every 4 weeks and analyzed at an age of 21 weeks. **(C)** FCM quantification of total splenic CD4^+^ T cell and B220^+^ B cell counts in 1×10^5^ spleen cells from MRL/*lpr* mice treated with pCAGGS-Mock or pCAGGS-Tgfb3 at an age of 21 weeks. **(D)** Estimated total splenic CD4^+^ T cell and B220^+^ B cell counts by multiplying each cell count by the compatible spleen weight. *P* < 0.05 by Student’s t-test. Error bars, s.d.

**
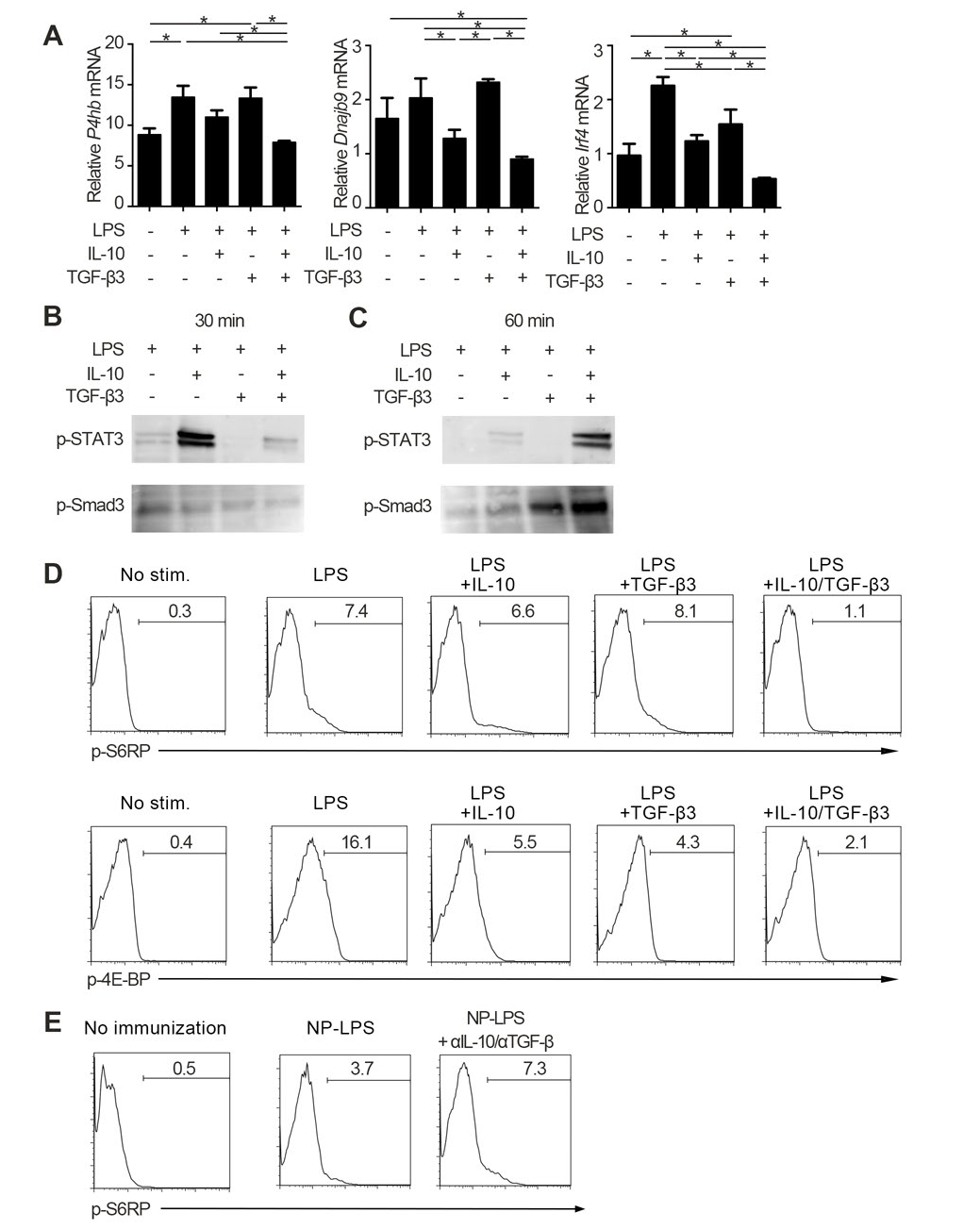
**

**FIGURE S4. TGF-β3 and IL-10 synergistically regulate gene expression profiles and downstream signaling in LPS-stimulated B cells. (A)** B cells were cultured under LPS stimulation either with TGF-β3 and/or IL-10 for 48-72 hr and the expression of the indicated genes was assessed by quantitative RT-PCR (*n* = 3). **(B, C)** Representative Western blot analyses of phosphorylated protein levels in LPS-stimulated B cells treated either with TGF-β3 and/or IL-10 for 30 min (B) and 60 min (C). **(D)** Representative histogram plots of phosphorylated S6RP at Ser235/236 and 4E-BP1 at Thr37/46 in 7-AAD negative LPS-stimulated B cells either with or without TGF-β3 and/or IL-10 for 72 hr. **(E)** Representative histogram plots of phosphorylated S6RP at Ser235/236 in splenic B220^+^ cells from NP-LPS-immunized B6 mice treated with anti-IL-10 antibody and anti-TGF-β antibody. *P* < 0.05 by one-way ANOVA followed by Bonferroni’s multiple comparisons test. Error bars, s.d..


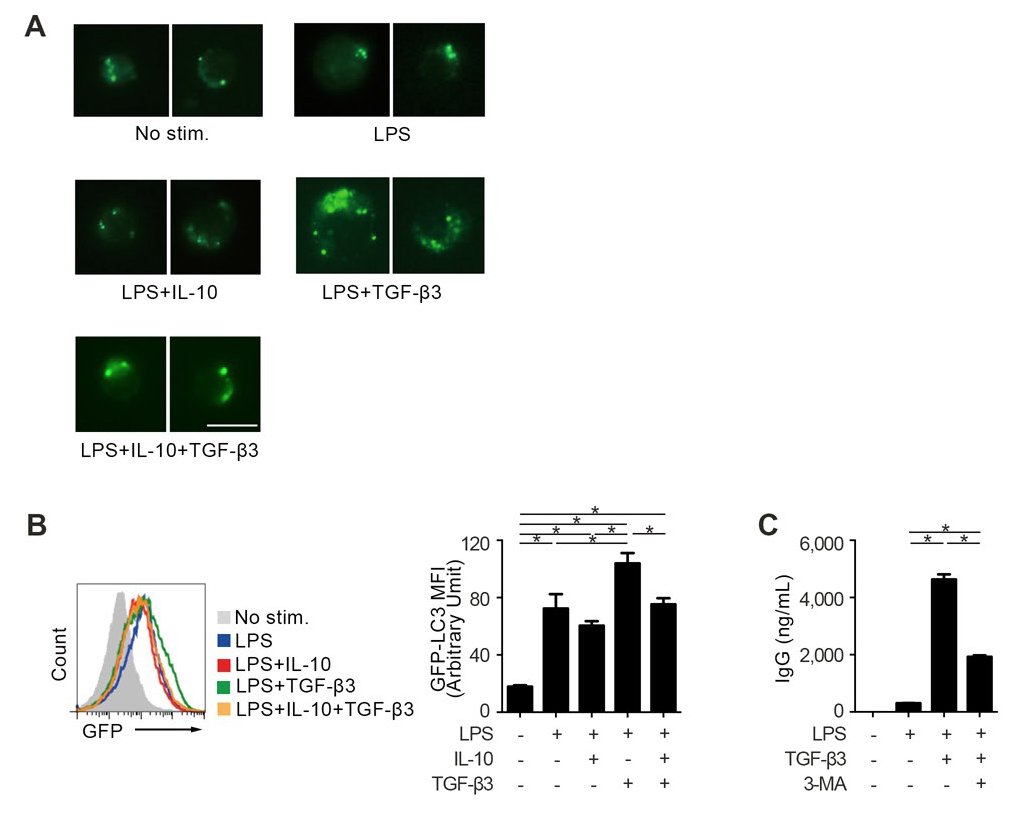


**FIGURE S5. Enhanced immunoglobulin production mediated by TGF-β3 is partially induced by increased autophagy. (A)** LC3-GFP mice splenic B cells were stimulated by LPS either with TGF-β3 and/or IL-10 for 3 days. After treatment, cells were washed with 0.05% saponin for 3 min and fixed for 10 min at room temperature with 4% paraformaldehyde. Images were captured using fluorescence microscopy. Scale bars, 10 μm.  **(B)** LC3-GFP mice splenic B cells were cultured for 3 days. Cells were treated with 100μM chloroquine last 4 hr and washed with 0.05% saponin for 3 min. Autophagosome-associated GFP-LC3-II in 7AAD^-^ cells were analyzed with flow cytometry (*n* = 3). **(C)** B cells from B6/J mice were cultured under LPS stimulation with TGF-β3 and 0.5mM 3-MA for 7 days. Total IgG production in the supernatants was determined by ELISA (*n* = 3). *P* < 0.05 by one-way ANOVA followed by Bonferroni’s multiple comparisons test. Error bars, s.d..


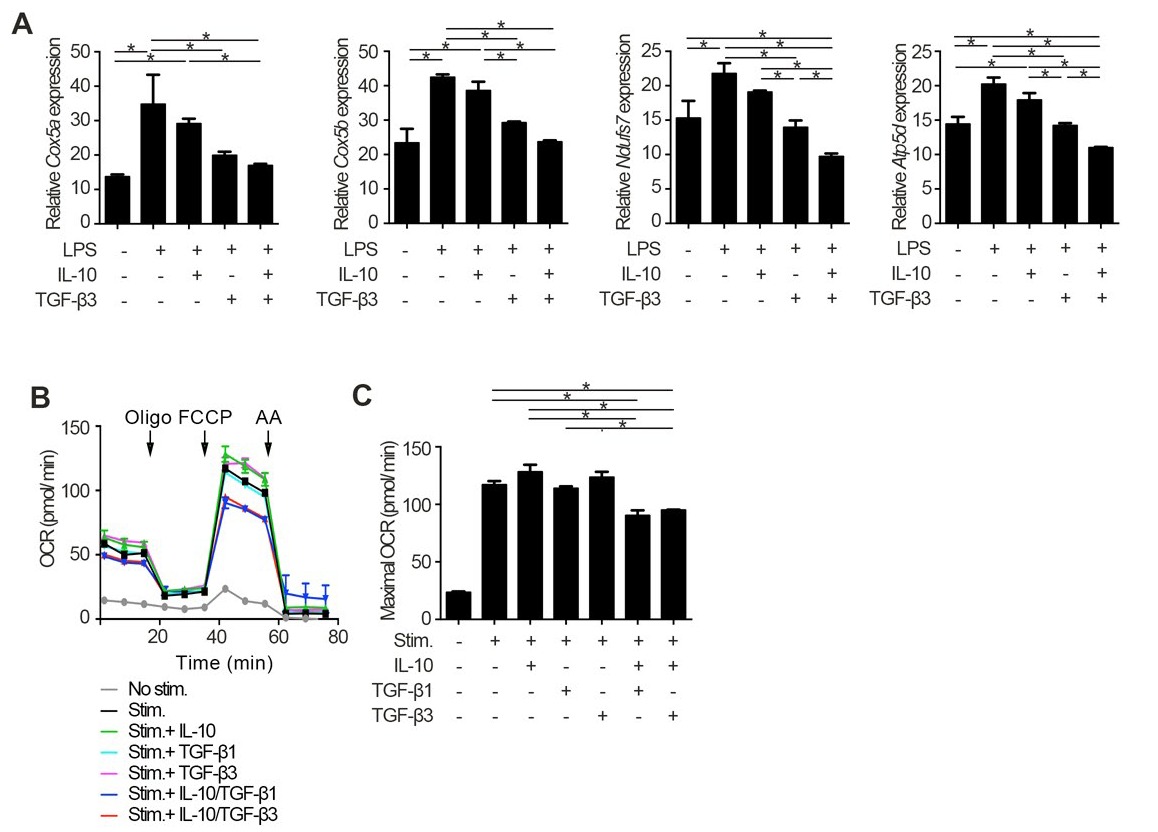


**FIGURE S6. TGF-β3 and IL-10 synergistically regulate B cell metabolism.** **(A)** B cells were cultured under LPS stimulation either with TGF-β3 and/or IL-10 for 24 hr and the expression of the indicated genes was assessed by quantitative PCR (*n* = 3). **(B, C)** Human B cells were stimulated by 2.5 μg/mL CpG-ODN, 1000 U/mL IL-2, 10 ng/mL IL-6, and 0.5 μg/mL anti-CD40 with 10 pg/ml TGF-β1, 10 pg/ml TGF-β3 and/or 10 ng/mL IL-10 for 3 d. OCR of human B cells were measured by extracellular flux analyzer (*n* = 3). *P* < 0.05 by one-way ANOVA (A, C) followed by Bonferroni’s multiple comparisons test. Error bars, s.d (A) and s.e.m. (B, C).
